# Conducting Polymer 3D Microelectrodes

**DOI:** 10.3390/s101210986

**Published:** 2010-12-03

**Authors:** Luigi Sasso, Patricia Vazquez, Indumathi Vedarethinam, Jaime Castillo-León, Jenny Emnéus, Winnie E. Svendsen

**Affiliations:** Department of Micro- and Nanotechnology, Technical University of Denmark, Ørsteds Plads 345ø, 2800 Kgs. Lyngby, Denmark; E-Mails: patricia.vazquez@nanotech.dtu.dk (P.V.); indumathi.vedarethinam@nanotech.dtu.dk (I.V.); jaime.castillo@nanotech.dtu.dk (J.C.-L.); jenny.emneus@nanotech.dtu.dk (J.E.); winnie.svendsen@nanotech.dtu.dk (W.E.S.)

**Keywords:** conducting polymers, micro-fabrication, micro-electrodes

## Abstract

Conducting polymer 3D microelectrodes have been fabricated for possible future neurological applications. A combination of micro-fabrication techniques and chemical polymerization methods has been used to create pillar electrodes in polyaniline and polypyrrole. The thin polymer films obtained showed uniformity and good adhesion to both horizontal and vertical surfaces. Electrodes in combination with metal/conducting polymer materials have been characterized by cyclic voltammetry and the presence of the conducting polymer film has shown to increase the electrochemical activity when compared with electrodes coated with only metal. An electrochemical characterization of gold/polypyrrole electrodes showed exceptional electrochemical behavior and activity. PC12 cells were finally cultured on the investigated materials as a preliminary biocompatibility assessment. These results show that the described electrodes are possibly suitable for future *in-vitro* neurological measurements.

## 1. Introduction

Electrical signals generated from neurons (action potentials) can be recorded with electrodes. These signals provide great insight about cell behavior, either as an individual neuron or as part of a network. Progress in technology has facilitated a journey that initiated with tissue-network response investigation [1,2], which currently is at the single cell level [3–5]. Microelectrode arrays (MEAs), normally in the form of planar electrodes, are used for simultaneous recording of signals from neuron cultures *in vitro*. However, the amplitude of these signals does not exceed 0.5 mV, which is poor compared to intracellular measurements, where the response lies in the range of 60–100 mV [6]. This is due to the fact that there is a physical distance between the cell and the surface of the electrode, which damps the recordings with unwanted noise. It is therefore crucial to use sensors that provide an excellent signal-to-noise ratio.

Needle-like electrodes with small tips offer the possibility to penetrate the cell and record intracellular activity [7]. Even for extracellular measurements this type of electrodes offers an improvement in the quality of the signal recorded; their higher surface area reduces the total impedance of the electrodes, which translates into a better signal-to-noise ratio. In the same way, studies of brain tissue can benefit from the use of out of plane electrodes. With larger dimensions than in the case of single cell analysis, they have been reported to improve the impedance of the recording system [8]. The signal quality is not only improved by the lower impedance of the electrodes; out of plane structures can penetrate the first dead cell layers of the tissue within the brain slice and reach the active neurons [9].

Different biocompatible materials and processes have been reported. They are typically used in the fabrication of Micro Electro Mechanical Systems (MEMS), like polydimethylsiloxane (PDMS) [10], SU8 photoresist [11], polyimide [12], glass [8,13] and silicon [9,14]. Despite the advantages, the use of three dimensional structures that are able to penetrate tissue is relatively new [8,9]. One of the main problems that hold back further development is the complexity of the fabrication process, which affects the cost and makes their production laborious and time consuming.

**Figure 1.**
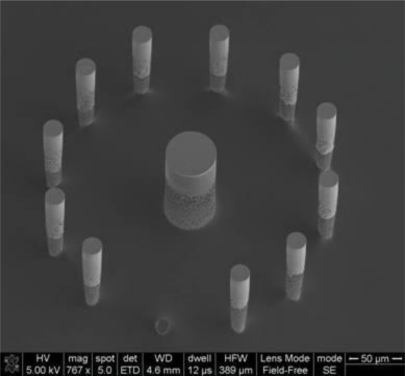
SEM image of electrodes partially metalized by sputtering. The image shows a clear example of how the walls of the electrodes are not covered by the metal layer at the bottom of the structures, which leaves them unconnected to the external wiring.

Sharp pillars can be etched in silicon using extensively studied methods such as wet and dry etch. However, these pillars need to become electrically active in order to perform as electrodes. The subsequent step of metallization of the electrode-pillars is a cumbersome task [15] and if done by traditional methods it renders the electrodes unconnected to the outside set up (Figure 1). Among the aforementioned methods, electrochemical deposition is not an option since there is not conductive layer existent yet in the electrodes substrate. More commonly used in microelectromechanical system (MEMS) fabrication are e-beam evaporation and sputtering of the surfaces in order to deposit a thin layer of metal onto them, but both techniques are insufficient to cover all the surface of very tall vertical walls (in the range of 70 μm or more). This is due to the fact that the angle of incidence of the ions of metal is far too narrow to reach the bottom of the vertical walls. Alternative methods and materials need to be found to tackle this problem, and they could be useful to ease the fabrication of similar structures that are found in MEMS devices. Such materials should be conductive and biocompatible with neurons to suit the final application of neurological studies.

After the original discovery of the electrically conductive properties of doped polyacetylene in 1977 [16], conducting polymers (CPs) have attracted much scientific attention during the subsequent decades. Interest in CPs has mainly arisen because of their high application potential. CPs can be used either as receptors, because of their affinity to inorganic ions, organic molecules and gases, or as transducers (optical or electrical) [17]. A crucial advantage of using a film of CP is the creation of a less polarizable surface compared to metal conductors.

Polyaniline (PAni) and polypyrrole (PPy) are CPs of special interest because of their wide range of applications, varying from microelectronics [18] to electrochemical sensors [19,20]. These materials are particularly used in biosensing applications because of their biocompatibility [21] and their ability for immobilizing biomolecules such as enzymes onto an electrode [22]. PPy has also been used in the past as electrode coating in order to stabilize semiconductor electrodes against photocorrosion in photoelectrochemical cells [23]. CPs can be synthesized both chemically [24] or electrochemically [22]. Although CPs’ electrochemical synthesis has extensively been investigated by the scientific community because of this technique’s advantages when it comes to adhesion to the underlying electrode, location-specific polymerization, thickness control and modulation of the polymer’s properties by changes in the electrochemical polymerization conditions, a chemical polymerization allows the formation of CP films onto non-conductive substrates. Chemical CP synthesis is a unique alternative when aiming at creating all-polymer devices, for example [25]. A main disadvantage of using chemical polymerization for the creation of thin CP films is the difficulty in processing such films onto specific structures.

Several micro-fabrication technologies have been used in the past for the patterning of chemically synthesized conducting polymers. By micromolding in capillaries, soft lithography allows the patterning of microstructues of conducting polymers with feature sizes ranging from 350 nm to 50 μm [26]. UV-lithography has been used to pattern spin-coated conducting polymer films into insulating and conducting sections of dimensions in the micron range [27]. Roll-to-roll nanoimprinting [28], inkjet printing [29] and a combination of nano-imprint lithography and isotropic plasma etching [30] have also been used for patterning. Shaping these materials into functional components remains a challenge because of their high melting points and very low solubility in common solvents. A novel patterning technique combining a chemical oxidative synthesis of the polymers with standard silicon fabrication technology has recently been developed [31].

In this article we present a combination of chemical polymerization methods and micro-fabrication techniques for creating conducting polymer pillar electrodes. Issues often arise when trying to metalize 3D structures by standard silicon microfabrication techniques because of poor metal coverage at the vertical side-walls of the structures. The chemical polymerization methods used in the work presented here offer a solution to this problem. The pillar structures can be coated with a thin film of conducting polymer by a chemical synthesis technique. Two different conducting polymers commonly used as sensor materials were used: polyaniline (PAni) and polypyrrole (PPy). Combinations of gold/conducting polymer electrodes were tested and characterized electrochemically, showing excellent behavior.

## 2. Fabrication

The fabrication process of the 3D structures has been described in detail in [15]. Briefly, the electrode-pillars were created as follows: a silicon wafer was spin coated with a 4.5 μm layer of AZ5214E photoresist, to be patterned with UV-lithography. A deep reactive ion etching technique was used to shape the micropillars, with a resulting height of 70 μm and diameters of 30, 50 and 70 μm. The electrodes were isolated from each other on the wafer with a 200 nm passivation layer of silicon nitride that was deposited by plasma enhanced chemical vapor deposition.

The metallization process, which is a major issue in this fabrication flow, was replaced by a polymerization of the wafer surface with a conductive polymer, as will be explained in detail below. This technique guarantees the conductivity of the electrodes, even in the case of poor coverage of their surface during a subsequent metallization. Such step is done after the polymerization in order to create the conductive paths for the external connections of the system. As a final step, another passivation layer was deposited in the same fashion as previously to protect the structures from a microfluidic environment and to control the electrode areas that are to be active. These areas are patterned by photolithography and exposed open with a deep reactive ion etch process.

### 2.1. Patterning of Conducting Polymer Film

**Figure 2.**
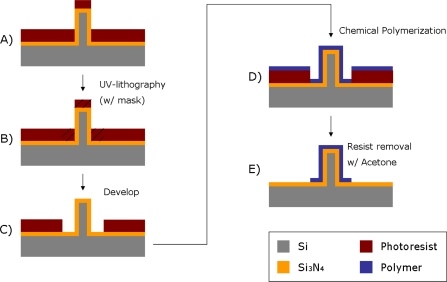
Schematic illustration of polymer film patterning. (**A**) Spin-coating of photoresist onto a silicon nitride surface previously deposited on a silicon wafer (containing the pillar structures); (**B**) Mask assisted UV-lithography; (**C**) Photoresist development; (**D**) Chemical synthesis of polymer film on the surface; (**E**) Sacrificial resist removal with acetone.

In this section a combination of chemical polymer synthesis and micro-patterning techniques is presented. The patterning was performed by UV-lithography using a photoresist as sacrificial material (Figure 2). With the use of a photolithographic mask, 40 μm wide lines were patterned on a silicon nitride surface previously spin-coated with photoresist on a silicon wafer.

**Figure 3.**
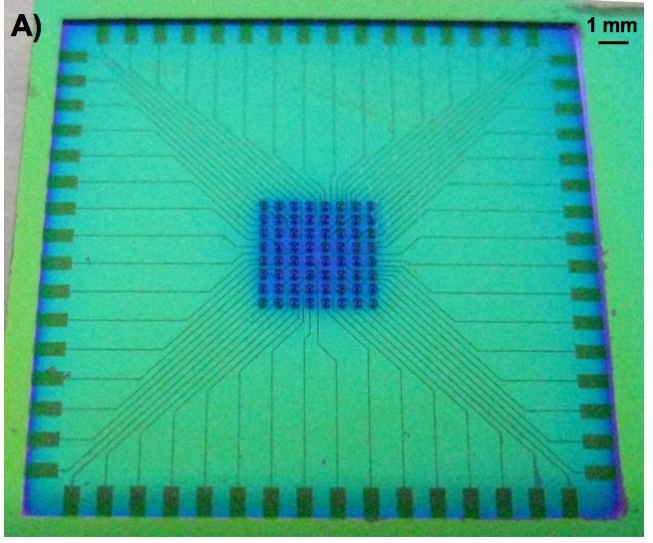

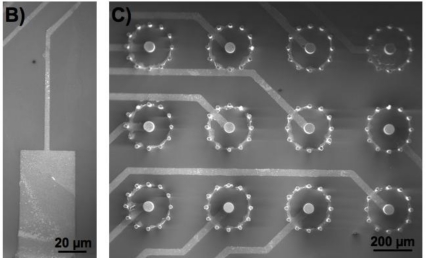
**(A**) Patterned polymer film on a silicon nitride surface (dark areas). The microchip included pads for electrical connections, conductive paths, and areas with 3D pillar electrode structures; (**B**) SEM top-view image of one of the pads; (**C**) SEM top-view image of areas with 3D microstructures. SEM images were obtained using a FEI Nova 600 NanoSEM system (FEI Company, OR, USA).

The wafer also held the 3-dimensional (3D) microstructures, or pillars, that are to be used as electrodes for biosensing applications. The 3D microelectrodes were placed in the center of each chip (dimension 2 cm × 2 cm). Each chip contained 64 electrodes, each individually addressable from its own contacting pad. The chips were composed of a silicon nitride background and three distinctive areas of conducting polymer (Figure 3). Around the edge of the microchip [Figure 3(A)], there were 64 0,5 mm × 1 mm pads [Figure 3(B)] to be used as electrical connections between the chip and external electrical components. Through a series of conductive paths (40 μm wide lines of polymer film), the pads were connected to 3D micropillars in the central area of the chip [Figure 3(C)].

The silicon nitride layer functions as an insulating material between the silicon wafer and the polymer film. Passivation of the pillars makes them isolated from each other on the silicon wafer. A 1.5 μm thick layer of photoresist was spin-coated onto the wafer containing the electrode structures, and a SEM analysis was carried out at the end of the process to confirm that the lithography was not affected by the presence of the pillar structures. The patterned areas of the photoresist were defined by UV exposure and development, which are standard techniques used in cleanroom lift-off processes. A mask was used containing alignment marks to ensure the exact location of the patterned areas. The surface was later immersed in a polymerizing solution (for details see below). The polymerizing solution was mixed quickly before the immersion, in order for the reaction to start only after the surface to be polymerized was already present in the solution. After having been left in the polymerizing solution for a defined time depending on the polymer used (10 min for polypyrrole synthesis, and 70 min for polyaniline synthesis) without stirring (in order for the polymerization products to sediment onto the surface), the sample was washed and dried with nitrogen gas. The polymer film was at this point present on all surfaces of the wafer, both exposed and unexposed areas were covered with photoresist. The sample was finally immersed in acetone for 15 min, in order for the sacrificial photoresist to dissolve, leaving behind patterned lines of the polymer thin-film reflecting the shape of the photolithographic mask used.

The main advantage for using a chemical oxidative reaction for the fabrication of polymer-based microelectrodes is that this method does not require a metal layer or any surface-catalyst to be present in the areas that are to be covered with polymer, as in the case of electropolymerization or electroless deposition [32]. The patterning of the polymer film is then done by a traditional sacrificial lithography technique, which does not require any etching steps, and is therefore also more cost effective. The experimental details for the formation of a PAni or PPy layer are slightly different since different conditions yield lead to better results for each of these materials (in terms of polymerization time and film thickness) [31], according to the following procedure: For the formation of a PAni layer, the polymerizing solution consisted of a combination of the monomer, aniline (Sigma-Aldrich, St. Louis, MO, USA), and ammonium persulfate (APS) (ICN Biomedicals, Inc., Aurora, OH, USA), an oxidizing agent needed for the chemical oxidative synthesis. A 10 mL solution containing APS (40 mM) in 1 M HCl (Sigma-Aldrich) was prepared. Aniline was added to a final concentration of 160 mM, and the solution was stirred. The samples, consisting of a silicon wafer covered with silicon nitride and a patterned layer of photoresist, were quickly immersed in the polymerizing solution and then left for 70 min unstirred. The samples were finally washed with 1M HCl and dried with nitrogen gas. The PPy synthesis was carried out in a similar way: A polymerizing solution was prepared by first dissolving 0.08 g of FeCl_3_ in 19 mL of double distilled (dd) water, and sequentially adding 300 μL of pyrrole monomer (Sigma-Aldrich). The silicon wafer was in this case immersed and left in the polymerizing solution for 10 min in order to achieve a polymer film of thickness in the order of 50 nm [31]. In this case the samples were washed with dd water and dried with nitrogen gas.

## 3. Cell Culturing

Untreated T25 flasks (NUNC, Thermo Scientific, Denmark) were coated with laminin (20 μg/mL, Sigma Aldrich, Denmark) in 1X Phosphate Buffered Saline (PBS) (Sigma Aldrich, Denmark) and left overnight. Later, excess laminin was removed and the flasks were washed twice with sterile water (Sigma Aldrich, Denmark). The Dulbecco’s Modified Eagle medium/Ham’s Nutrient Mixture F12 supplemented with 10% fetal bovine serum, 10% horse serum, 100 Units/mL penicillin and 100 μg/mL streptomycin and 25 mM HEPES (referred to as DMEM/F12 from now on) was added in the flasks and placed at 37 °C in humid atmosphere containing 95% air and 5% CO_2_.

Undifferentiated PC12 cells (PC12—pheochromocytoma of rat adrenal medulla, DSMZ GmbH, Germany) approximately 4 × 10^6^ cells were thawed and transferred to the above-mentioned flasks and placed in the incubator. After 5 days, neuronal differentiation was initiated by replacing the media with DMEM/F-12 supplemented with 100 ng/mL NGF. The cell media was changed every 2 days. After 5 days of culturing, the cells were detached by adding 0.05% trypsin-EDTA (Sigma Aldrich, Denmark) at 37 °C for 3 min. The cell suspension was transferred to a 10 mL Falcon tube with fresh media and centrifuged at 1,100 rpm for 3 min. The supernatant was removed and the pellet was resuspended with media at 1 × 10^6^ cells/mL. The centrifugation process and the resuspension of the cells in fresh media are done in order to remove the trypsin.

The differentiated cells were then seeded in a Petri dish containing the electrode chip and it was supplemented with DMEM/F-12 and 100 ng/ml NGF. Before seeding the cells, the electrode chips were sterilised with acetone at 70 °C for 5 min followed by three times wash in 70% ethanol for 5 min. The chips were washed with 1X PBS three times and then coated with laminin, as previously described.

After 5 days, the cells were fixed in 2% glutaraldehyde in 0.1 × PBS for an hour. The sample was rinsed in 0.1 × PBS for 15 min at 4 °C and washed with deionized water for 5 min. The sample was dehydrated with increased percentages of ethanol (50, 60, 70, 80, 90, and finally 100%) and freeze dried for 2 h followed by Scanning Electron Microscope (SEM) imaging.

## 4. Results and Discussion

**Figure 4.**
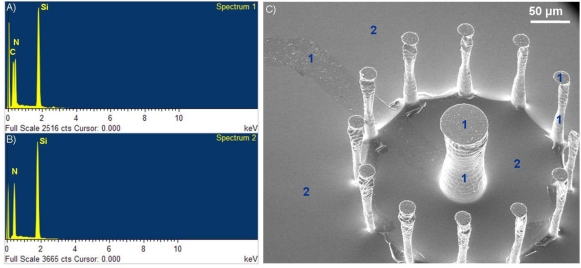
Validation of the presence of polymer films by EDX Analysis: (**A**) EDX spectrum of a polyaniline covered surface; (**B**) EDX spectrum of the silicon nitride surface without the PANI film; (**C**) SEM image of one of the 3D structures, tilted to a 30° angle, with corresponding areas: 1 (where the film was present, spectrum 1) and 2 (where the film was not present, spectrum 2).

Validation of the presence of the polymer film on the surface of the microchip was achieved through Energy Dispersive X-ray (EDX) analysis (Oxford Inca EDX system, Oxford Instruments, United Kingdom). In Figure 4, two spectra are shown pertaining to areas of the surface where a PAni film synthesized with the methods described above are present [Figure 4(A)] and not [Figure 4(B)]. The presence of polymer on the surface is characterized by an elemental carbon peak at around 0,23 keV, on the horizontal axis of the EDX spectra. This same carbon peak is not present in areas of the surface that were covered by photoresist during the patterning process. It is important to note that the polymer film was present not only on the 2D areas of the surface, but also on the walls of the 3D micropillars for both the PAni and PPy case. Adhesion of the film onto vertical walls makes it possible for these conductive materials to be used as an alternative or addition to metal layers that usually are deposited by sputtering, a process which is directionally oriented and therefore does not yield good results on surfaces parallel to the sputtering direction.

The thickness of the polymer film was measured with a surface profiler (Dektak 8 Advanced Development Profiler system, Veeco, Plainview, NY, USA) by scanning areas containing the 40 μm film stripe along the direction of the stripe’s width. The obtained height vs. distance plot is a good illustration of the presence of the polymer film. The thickness of the PAni film was averaged from this data to be 90.6 ± 6,7 nm, while the average thickness of the PPy film was 53.2 ± 8.1 nm. Both thickness values agree with the film thicknesses expected for the specific conditions used during the chemical polymerization [31].

The use of PAni as a coating for the 3D structures showed good results as for the methodology, but PAni lacks good conductive properties in the context of cell signaling, where an excellent sensitivity is required. This fact was corroborated by electrochemical characterization of the PAni electrodes. Because of the nature of the material, PAni is only conductive at low pHs [33]. The acidic conditions needed to utilize the PAni electrodes do not constitute an environment that is compatible with cell culturing, which makes this conducting polymer unsuitable for neurological applications. Taking a step further into the investigation of conducting polymers, PPy was introduced as a possible alternative to PAni due to the broader range of environmental conditions that still allows high conductivity of the material.

**Figure 5.**
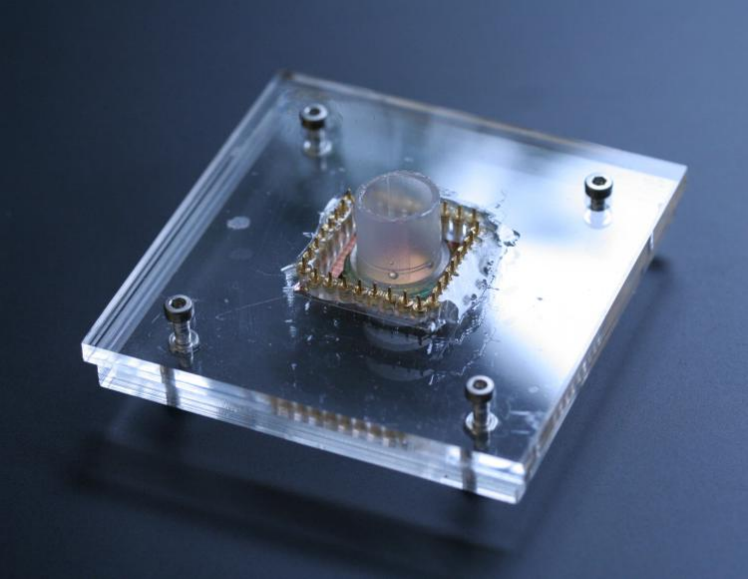
PMMA holder used for electrochemical experiments.

The electrochemical activity of the PPy coated pillar electrodes was characterized by cyclic voltammetry. A plastic gasket was glued on the surface of the chip containing the electrodes so as to create a liquid container for the electrochemical experiments. A holder was fabricated in Poly(methyl methacrylate) (PMMA) using micromilling techniques to facilitate the experimental handling [see Figure (5)]. Connections between the potentiostat and the chip were ensured using spring-contacts embedded in the holder. A 3-electrode system was used for the cyclic voltammetry experiments, where the 3D microelectrodes served as the working electrodes, an Ag/AgCl (3M KCl) as reference and a Pt wire as counter electrode. A ferrocyanide solution (10 mM) in PBS (pH 7.4) was used as the electrolyte, and the potential was cycled from −0.2 to 1.0 V at several potential sweep rates in the range of 50−500 mV/s.

**Figure 6.**
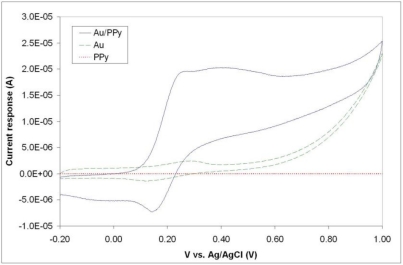
Cyclic voltammograms of single 3D microelectrodes in Au, PPy, and Au/PPy combination, obtained in a 10 mM Ferrocyanide aqueous solution in PBS (pH 7.4), using a Ag/AgCl (3M KCl) reference electrode and a Pt wire counter electrode. Voltage is cycled from −0.2 to 1.0 V with a potential sweep rate of 200 mV/s.

Figure 6 illustrates typical cyclic voltammograms of microelecrodes in PPy, Au, and Au/PPy, obtained with a Reference 600 potentiostat/galvanostat/ZRA (Gamry Instruments, Warminster, PA, USA). It is evident from the voltammograms that the PPy film alone does not exhibit a sufficiently high current response. Since the conducting polymer film alone does not yield a high-quality electrochemical activity; a hybrid combination consisting of 200 nm gold layer covered with a thin polypyrrole film was investigated. The fabrication of such structures includes a metallization step and consecutively the chemical polymerization process described in Section 2.1, in order to form first a layer of metal and secondly a polymer coating. The same patterning procedure described earlier was carried out before both steps. SEM and EDX analyses confirmed the presence of both gold and polypyrrole on the exposed patterned areas of the surface. A comparison of the voltammograms shows that the inclusion of the polymer film increases the current response of the electrodes. This increase is thought to be primarily due to fabrication issues connected with the metallization process. The electron-beam evaporation technique used to deposit the metal layer has typically poor side-wall coverage [see Figure (1)], meaning that the electrode’s surface area obtained with this technique is not in agreement with the geometrical surface area of the pillar. We hypothesize that the polymer film is able to bridge the gaps created by the poor metallization on the vertical walls of the electrode structures. The inclusion of the conducting polymer film therefore ultimately increases the active electrode area. Without the polymer, the voltammogram seen in Figure 6 reflects the current response of the relatively small gold surface area present at the bottom of the pillar structure, *i.e*., the connection between the planar conductive paths and the 3D electrode structure [see Figure 3(C)]. The conducting polymer ensures that all the surface area of the electrode is used. We can conclude from this investigation that the electrochemical behavior of the hybrid Au/PPy electrodes is the one best suited for our application because of its higher current response. Although no signs of detachment of the conducting polymer films were observed during the characterization, the adhesion properties of chemically synthesized polypyrrole films should be further investigated, as this is a major factor in electrode stability during actual in-vitro measurements when live cells are present in the system.

**Figure 7.**
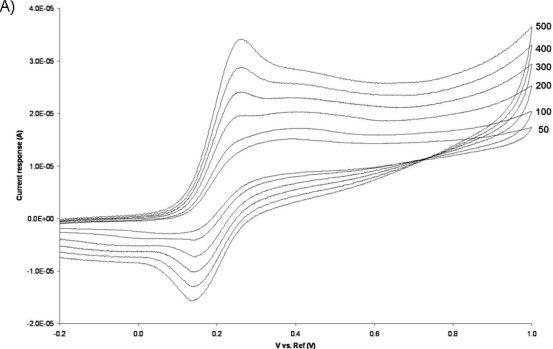

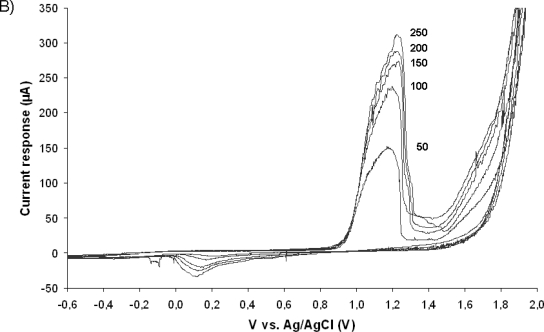
Typical cyclic voltammetric behavior of a single 3D Au/PPy microelectrode, using a Ag/AgCl (3M KCl) reference electrode and a Pt wire counter electrode, obtained in (A) a 10 mM Ferrocyanide aqueous solution in PBS (pH 7.4) where the voltage is cycled from −0.2 to 1.0 V for several potential sweep rates between 50 and 500 mV/s, and (B) a 0.1 M KCl solution in double distilled water with potential sweep rates of 50, 100, 150, 200 and 250 mV/s in a potential window between −0.6 and 2.0 V. Insets for both plots show the linear relationship between current peak response and square root of the potential sweep rate.

Electrochemical characterization of the Au/PPy electrodes was carried out by cyclic voltammetry in a 10 mM Ferrocyanide solution in PBS. The obtained voltammograms for several different potential sweep rates can be seen in Figure 7A. The Au/PPy electrodes exhibit a proportional increase in the current peak response with the square root of the potential sweep rate [see inset in Figure 7(A)], which is a sign of reversibility. The anodic peaks correspond to the ferrocyanide oxidation to ferricyanide, and the cathodic peaks to the reverse reaction, *i.e.*, the reduction of ferricyanide to ferrocyanide. In this set of experiments the Au/PPy electrode acts as a site for electron transfer. In order to view the redox behavior of the polymer layer itself, using the gold layer surface as the electron transfer site and the polymer film as a solid electrolyte, cyclic voltammetry experiments were carried out in a 0.1 M KCl solution in double distilled water. As shown in Figure 7B, clear anodic and cathodic peaks are present. Again, a linear increase in current response is seen with the square root of the potential sweep rate [see inset in Figure 7(B)]. The Au/PPy electrodes show anodic peak potentials at around 1.2 V and cathodic peaks at around 0.1 V (*vs.* Ag/AgCl). This voltammetric behavior is similar to that of electropolymerized polypyrrole films obtained by cyclic voltammetry on conventional metal electrodes [34].

**Figure 8.**
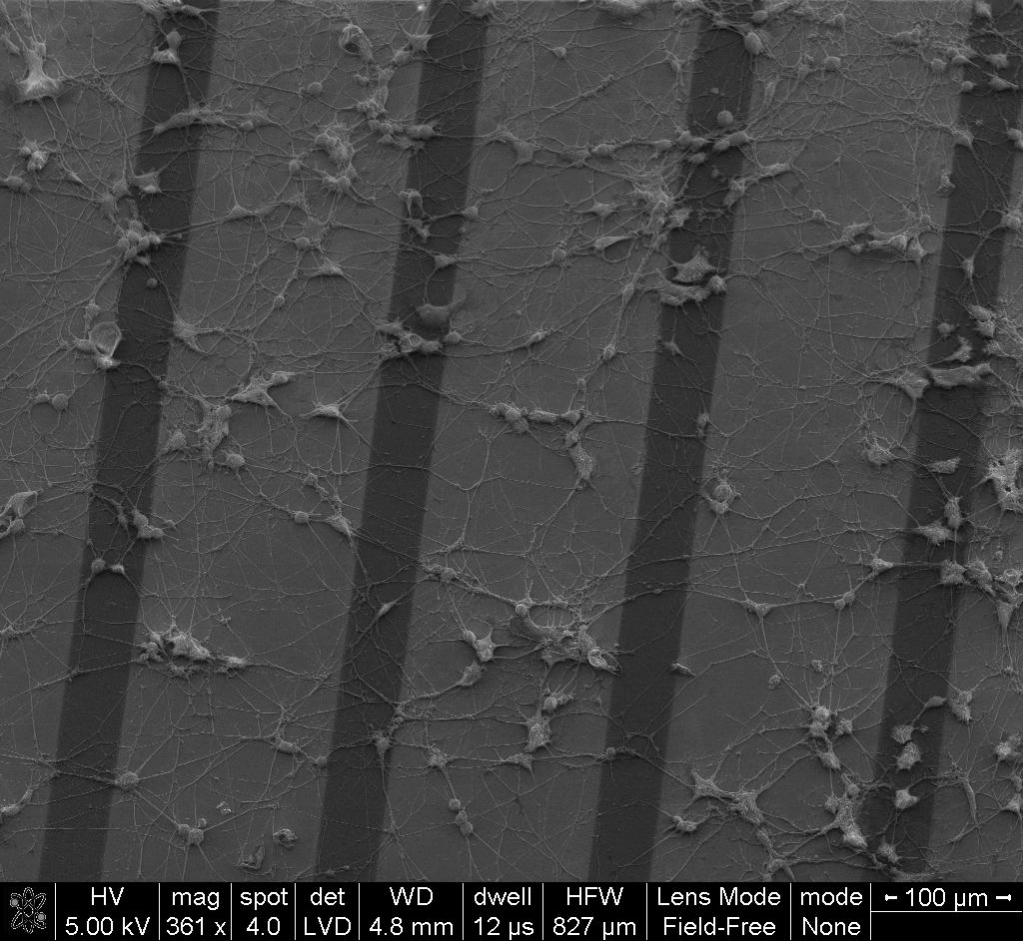
SEM image of PC12 cells cultured on the Au/PPy electrode chip.

PC12 cells are widely used as neuron model system. In this study PC12 cells were cultured on the Au/PPy electrode chips in order to investigate the feasibility of using the described electrodes for cellular and tissue studies, mainly out of concern for possible toxicity effects due to desorption of left-over monomers in the polypyrrole film. The cultures were maintained for 5 days on the chips. SEM images indicate the typical morphological features of differentiated PC12 cells. A similar cell growth pattern could be seen on all parts of the chips (see Figure 8).

The neuronal model cells showed a cellular morphology and adhesion on the electrode chips typical of PC12 cells [35]. By these preliminary results, we concluded that the Au/PPy electrode chips could potentially be used for neurological applications.

## 5. Conclusions

In summary, we have produced conducting polymer 3D microelectrodes fabricated in silicon and coated with polyaniline and polypyrrole films using a combination of micro-fabrication techniques and chemical polymerization methods. The thin films of conducting polymers achieved were uniform and adhered well to both horizontal and vertical surfaces. A comparison of electrodes in polypyrrole, gold, and gold/polypyrrole was made through cyclic voltammetry. Superior results were obtained for hybrid Au/PPy electrodes. The presence of the polymer film drastically increased the otherwise relatively low current response during voltammetry experiments. An electrochemical characterization of such electrodes showed excellent electrochemical behavior and activity. PC12 cells were cultured on the Au/PPy electrode chips to exclude possible toxicity effects of the materials. Future work will focus on utilizing the electrode presented in this article for *in-vitro* neurological measurements, comprising of thorough biocompatibility assessments of the described structures and materials, stability studies of the polymer films under cells and tissue culturing environments, and finally electrochemical measurements of triggered neurotransmitter releases (*i.e*., dopamine).

## References

[1] Thomas CA, Springer PA, Loeb GE, Berwald-Netter Y, Okun LM (1972). A miniature microelectrode array to monitor the bioelectric activity of cultured cells. Exp Cell Res.

[2] Gross GW, Rieske E, Kreutzberg GW, Meyer A (1977). A new fixed-array multi-microelectrode system designed for long-term monitoring of extracellular single unit neuronal activity *in vitro*. Neurosci Lett.

[3] Maher MP, Pine J, Wright J, Tai Y-C (1999). The neurochip: A new multielectrode device for stimulating and recording from cultured neurons. J Neurosci Meth.

[4] Xiang G, Pan L, Huang L, Yu Z, Song X, Cheng J, Xing W, Zhou Y (2007). Microelectrode array-based system for neuropharmacological applications with cortical neurons cultured *in vitro*. Biosens Bioelectron.

[5] Greve F, Lichtenberg J, Kirstein K-U, Frey U, Perriard J-C, Hierlemann A (2007). A perforated CMOS microchip for immobilization and activity monitoring of electrogenic cells. J Micromech Microeng.

[6] Ionescu-Zanetti C, Shaw RM, Seo JG, Jan YN, Jan LY, Lee LP (2005). Mammalian electrophysiology on a microfluidic platform. Proc Nat Acad Sci USA.

[7] Huys R, Braken D, Van Meerbergen B, Winters K, Eberle W, Loo J, Tsvetanova D, Chen C, Severi S, Yitzchaik S, Spira M, Shappir J, Callewaert G, Borghs G, Bartic C (2008). Novel concepts for improved communication between nerve cells and silicon electronic devices. Solid State Electron.

[8] Heuschkel MO, Fejtl M, Raggenbass M, Bertrand D, Renaud P (2002). A three-dimensional multi-electrode array for multi-site stimulation and recording in acute brain slices. J Neurosci Meth.

[9] Thiebaud P, Beuret C, de Rooij NF, Koudelka-Hep M (2000). Microfabrication of Pt-tip microelectrodes. Sensors Actuat B-Chem.

[10] Pérennès F, Marmiroli B, Matteucci M, Tormen M, Vaccari L, Di Fabrizio E (2006). Sharp beveled tip hollow microneedle arrays fabricated by LIGA and 3D soft lithography with polyvinyl alcohol. J Micromech Microeng.

[11] Rajaraman S, Choi S-O, Shafer RH, Ross JD, Vukasinovic J, Choi Y, DeWeerth SP, Glezer A, Allen MG (2007). Microfabrication technologies for a coupled three-dimensional microelectrode, microfluidic array. J Micromech Microeng.

[12] Chen Y-Y, Lai H-Y, Lin S-H, Cho C-W, Chao W-H, Liao C-H, Tsang S, Chen Y-F, Lin S-Y (2009). Design and fabrication of a polyimide-based microelectrode array: Application in neural recording and repeatable electrolytic lesion in rat brain. J Neurosci Meth.

[13] Lin C-W, Lee Y-T, Chang C-W, Hsu W-L, Chang Y-C, Fang W (2009). Novel glass microprobe arrays for neural recording. Biosens Bioelectron.

[14] Hanein Y, Lang U, Theobald J, Wyeth R, Daniel T, Willows AOD, Denton DD, Bohringer KF (2001). Intracellular neuronal recording with high aspect ration MEMS probes. Transducers ′01: Eurosensors XV, Digest of Technical Papers, Vols 1 and 2.

[15] Vazquez P, Dimaki M, Svendsen WE (2009). Metallization of High Aspect Ratio, out of Plane Structures. Proceedings of 3rd International Workshop on Advances in Sensors and Interfaces.

[16] Chiang CK, Fincher CR, Park YW, Heeger AJ, Shirakawa H, Louis EJ, Gau SC, MacDiarmid AG (1977). Electrical conductivity in doped polyacetylene. Phys Rev Lett.

[17] Lange U, Roznyatovskaya NV, Mirsky VM (2008). Conducting polymers in chemical sensors and arrays. Anal Chimica Acta.

[18] Bruce PG, McGregor ES, Vincent CA (1992). The perfectly polarized polymer electrolyte/electrode interface. Electrochimica Acta.

[19] Chao S, Wrighton MS (1987). Solid-state microelectrochemistry—electrical characteristics of a solid-state microelectrochemical transistor based on poly(3-mehylthiophene). J Am Chem Soc.

[20] Ayad MM, Salahuddin NA, Alghaysh MO, Issa RM (2010). Phosphoric acid and pH sensors based on polyaniline films. Curr Appl Phys.

[21] Janata J, Josowicz M (2003). Conducting polymers in electronic chemical sensors. Nat Mater.

[22] Hangarter CM, Bangar M, Mulchandani A, Myung NV (2010). Conducting polymer nanowires for chemiresistive and FET-based bio/chemical sensors. J Mater Chem.

[23] Bartlett PN, Cooper JM (1993). A review of the immobilization of enzymes in electropolymerized films. J Electroanal Chem.

[24] Skotheim T, Petersson LG, Inganas O, Lundstrom I (1982). J Electrochem Soc.

[25] Rapi S, Bocchi V, Gardini GP (1988). Conducting polypyrrole by chemical synthesis in water. Synthetic Met.

[26] Voss D (2000). Cheep and cheerful circuits. Nature.

[27] Beh WS, Kim IT, Qin D, Xia Y, Whitesides GM (1999). Formation of patterned microstructures of conducting polymers by soft lithography, and applications in microelectronic device fabrication. Adv Mater.

[28] Mäkelä T, Pienimaa S, Jussila S, Isotalo H (1999). Lithographic patterning of conducting polyaniline. Synthetic Met.

[29] Mäkelä T, Haatainen T, Majander P, Ahopelto J (2007). Continuous roll to roll nanoimprinting of inherently conducting polyaniline. Microelectron Eng.

[30] Crowley K, Morrin A, Smyth MR, Killard AJ, Shepherd R, in het Panhuis M, Wallace GG (2008). Fabrication of chemical sensors using inkjet printing and application to gas detection. IEEE Sens J.

[31] Huang C, Dong B, Lu N, Yang B, Gao L, Tian L, Qi D, Wu Q, Chi L (2009). A strategy for patterning conducting polymers using nanoimprint lithography and isotropic plasma etching. Small.

[32] Dong B, Zhong Dingyong, Chi L, Fuchs H (2005). Patterning of conducting polymers based on a random copolymer strategy: Toward the facile fabrication of nanosensors exclusively based on polymers. Adv Mater.

[33] Attout A, Yunus S, Bertrand P (2008). Electroless deposition of polyaniline: Synthesis and characterization. Surf Interface Anal.

[34] MacDiarmid AG (2001). Synthetic metals: A novel role for organic polymers (Nobel Lecture). Angew Chem Int Ed.

[35] Sayyah SM, El-Rehim SSA, El-Deep MM (2003). Electropolymerization of pyrrole and characterization of the obtained polymer films. J Appl Polymer Sci.

[36] Freshney RI (2005). Culture of Animal Cells: A Manual of Basic Technique.

